# A method for validating Rent’s rule for technological and biological networks

**DOI:** 10.1038/s41598-017-05670-w

**Published:** 2017-07-14

**Authors:** Fernando Alcalde Cuesta, Pablo González Sequeiros, Álvaro Lozano Rojo

**Affiliations:** 1 GeoDynApp - ECSING Group, Santiago de Compostela, Spain; 2 Departamento de Matemáticas, Universidade de Santiago de Compostela, E-15782 Santiago de Compostela, Spain; 3 Departamento de Didácticas Aplicadas, Facultade de Formación do Profesorado, Universidade de Santiago de Compostela, Avda. Ramón Ferreiro s/n, E-27002 Lugo, Spain; 4 Centro Universitario de la Defensa Zaragoza, AGM, Ctra. Huesca s/n, E-50090 Zaragoza, Spain; 5 Instituto Universitario de Matemáticas y Aplicaciones, Universidad de Zaragoza, E-50009 Zaragoza, Spain

## Abstract

Rent’s rule is empirical power law introduced in an effort to describe and optimize the wiring complexity of computer logic graphs. It is known that brain and neuronal networks also obey Rent’s rule, which is consistent with the idea that wiring costs play a fundamental role in brain evolution and development. Here we propose a method to validate this power law for a certain range of network partitions. This method is based on the bifurcation phenomenon that appears when the network is subjected to random alterations preserving its degree distribution. It has been tested on a set of VLSI circuits and real networks, including biological and technological ones. We also analyzed the effect of different types of random alterations on the Rentian scaling in order to test the influence of the degree distribution. There are network architectures quite sensitive to these randomization procedures with significant increases in the values of the Rent exponents.

## Introduction

Rent’s rule was described by Landman and Russo^1^ in 1971 from two IBM internal memoranda by Rent in 1960. It relates the average number of external connections or *pins* on a module and the average number of blocks within the module for partitions of computer logic graphs^2^. Besides its extensive application in Circuit Design at all scales, from SSI to GSI passing through VLSI, Rent’s rule has been used to study the interconnection complexity of biological networks^3–6^, as well some benchmark models and technological networks^7–9^. At origin, given a logic circuit, the relationship between the average number of blocks or cells *B* in a module in a given partition and the average number of pins *P* connecting each module with the others is

1 $$P=k{B}^{p}$$

where *k* is the average number of pins per logic block (also called *Rent coefficient*) and *p* is the *Rent exponent* describing proportionality in a log-log scale. As Landman and Russo experimentally observed, there is an empirical confirmation of this power law in a certain region, called *Region I*, but Rent’s rule overestimates the interconnection complexity of the circuit in another region, called *Region II*, where the number of modules is small^1^. Usual approaches of calculating Rent exponents are based on partitioning of the logic (hyper)graph. Although it could be possible that the partitioning-based algorithm itself caused Region II to appear, it also appears for other algorithms partitioning-based^10^, placement-based^11^ and spectral-based^4^. See refs 11 and 12 for a comparative analysis of Rent parameters extracted from partitioning-based and placement-based algorithms. On the other hand, it was also observed by Stroobandt^10^ that Rent’s rule underestimates the interconnection complexity of some circuits when the number of modules is large, leading to a new *Region III*. Anyway, the exact value of the Rent exponent and the transitions from Region I to Regions II and III depend on the partitioning method.

In this paper, we propose a method to determine Region I where experimental data fit well to Rent’s rule (1). Since VLSI circuits are disposed in the plane, it would be reasonable to derive Rent’s rule from placement-based algorithms making use of the geometrical information of the placed circuit. A similar argument could be applied to some networks studied in a previous paper^9^, namely the *US Power Grid* network^13^ and the *Internet2* academic network. However, it does not apply to neural networks, like that of the nematode *Caenorhabditis elegans* ^5, 9^, hierarchical modular graphs^8, 9^ or geometrical random graphs^8, 9^. Accordingly, we adopt a topological point of view, which is also present in other authors’ works^5, 8, 9^, where Rent’s rule is directly deduced from graph partitioning without distinguishing where and how it is embedded. As explained by Bassett *et al*.^5^, Rentian scaling is now related to the fractal dimension of the graph^14, 15^. Even if this approach is often illustrated with a wrong image representing the network embedded into the plane, the traditional recursive bipartitioning is consistent with this topological perspective^2, 11^. However, despite some limitations (caused by low diameters in the graphs and random choices in the partitioning processes), we preferred the box counting method by Song *et al*.^14–16^ that we have already used^9^. More precisely, we implemented the greedy coloring algorithm proposed by Song *et al*.^16^ according to the description given by Locci *et al*.^17^. We tested our method on some technological and biological networks (Internet2^9^, US Power Grid^13^, *C. elegans* connectome^18–20^ and Yeast interactome^21^) and some benchmark VLSI circuits (s838, s9234, s953, and c5315) from the ISCAS89 suite^22^, which have already been analyzed by other authors. We also added the VLSI circuit ibm01 from the ISPD98 suite^23^. The exact values of the Rent exponents have been compared with those obtained by these authors.

General models for VLSI circuits and neural networks actually consist of hypergraphs where an edge can connect more than two nodes^6, 24^. Special versions of partitioning-based algorithms, such as those used in the partitioning tool hMetis^25^, have been developed for hypergraphs. However, even if the considerations by Partzsch *et al*.^6^ should be taken into account, we transformed hypergraphs into graphs in order to unify the framework. The scaling curves obtained via hMetis show a very different behavior of those obtained via box counting since Regions II and III are very small if present. They also show a very different behavior with respect to the randomization process used for validating Rent’s rule (1). But both behaviors are consistent in the sense that Rentian scalings seem to respond to certain features in the network architecture, at least in restriction to Region I, independently of the partitioning algorithm used. A possible link with degree distribution has been tested on three different types of random networks: a *configuration model* with the same degree distribution as each empirical network^26, 27^, a configuration model without multiple or self-connections according to *Chung-Lu variant* ^28^, and a *Erdös-Rényi model* exhibiting another degree distribution. For each empirical network, our experiments proved that, independently of whether the degree distribution is preserved or not, all these random models have very similar Rent characteristics when hMetis is used. However, when the box counting method is applied, the influence of each specific network architecture is clearly visible in the transitions between Regions I and II, while some analogies between Rentian scalings persist in restriction to the corresponding Regions I.

## Validating Rent’s rule

As we previously said, deviations from Rent’s rule (1) usually appear at high and low values of *B* defining Region II and Region III respectively. For each network, there is an empirical discrete function *P* = *P*_*c*_(*B*), called *Rent characteristic* ^6, 7^, which directly relates the average number of nodes *B* for a topological partition of the network to the average number of edges *P* connecting different modules of the partition. We are interested in determining Region I where Rent characteristic fits well to Rent’s rule.

### Network data

The US Power Grid (PG) network is the high-voltage power grid in the Western States in the USA^13^. The nodes are generators, transformers, or substations, and the edges are high-voltage transmission lines. Originally used by Watts and Strogatz^29^, this undirected network has 4,921 nodes and 6,594 edges.

The Internet2 (I2) network collects data from Internet2 community, available now through the Global Research Network Operations Center (GlobalNOC) at Indiana University^30^, but which were originally collected in April 2013. For more details, see Supplementary Information of ref. 9. This network has 311 nodes and 323 edges.

The *C. elegans* neuronal (CE) network incorporates data from ref. 18 and updates based upon refs 19, 20. In the version published by Varshney *et al*.^31^, the *C. elegans* connectome has 279 somatic neurons, 6,393 chemical synapses, 890 gap junctions, and 1,410 neuromuscular junctions. We do not distinguish directionality of connections in this network that combines undirected gap junctions with directed chemical synapses. We also ignore neuromuscular junctions and synaptic multiplicities. Thus, all the unidirectional connections between two different neurons will be replaced by bidirectional ones leading to a total of 279 nodes connected by 2,290 edges. Since neurons RIBL/R and VA08 have auto-connections, we restrict our attention to 2,287 connections, cf. refs 5, 20, 32. Different values for its Rent exponent has been already reported by other authors^3, 5, 6^.

The Yeast Protein-Protein Interaction (Y2) network has been downloaded from the Yeast Interactome Project at the Center for Cancer Systems Biology^33^. As explained by Yu *et al*.^21^, CCSB-YI1 dataset containing high-quality yeast two-hybrid protein-protein interactions for *S. cerevisiae* ^21^ has been combined with Ito-core and Uetz-screen datasets to produce Y2H-union. This dataset contains 2,930 interactions among 2,018 proteins, which approximately represent 20% of the whole yeast interactome, but we have limited ourselves to the largest connected component made of 2,518 interactions among 1,647 proteins. A former version of the yeast interactome was studied by Reda^3^.

Some benchmark VLSI circuits from the ISCAS89 suite^22^ have been also considered. The Rent exponents of the VLSI benchmarks s838 and s9234 were computed by Reda^3^, and that of s953 by Bassett *et al*.^5^. The Rent characteristic of s953 and c5315 are described by Stroobandt^10^. We added the VLSI circuit ibm01 from the ISPD98 suite^23^, which was included in some experiments by Verplaetse *et al*.^34^. The order *N* and size *E* of all these graphs are reported in Table 1.

**Table 1:** Rent exponents for different partitioning algorithms and different thresholds compared with values obtained by other authors^3, 5, 6, 34^.

Network	N	E	*c* ≥ 0.1		*c* ≥ 0.075		hMetis	3	5	6	34	34 optimized
			No Reg. III	Δ > 10^−3^	No Reg. III	Δ > 10^−3^						
PG	4,941	6,594	0.712^***^	**0.743** ^***^	0.712^***^	**0.743** ^***^	0.730^***^					
I2	311	323	**0.689** ^***^	**0.689** ^***^	**0.689** ^***^	**0.689** ^***^	0.517^***^					
CE	279	2,287	0.750^**^	0.750	**0.916** ^**^	**0.916**	0.671^***^	0.781	0.740	0.827		
Y2	1,647	2,518	0.918^***^	**0.954** ^***^	0.918^***^	**0.954** ^***^	0.759^***^	0.489				
s838	457	702	**0.793** ^***^	**0.793** ^***^	**0.793** ^***^	**0.793** ^***^	0.520^***^	0.367				
s9234	5,809	8,165	0.797^***^	**0.838** ^***^	0.797^***^	**0.838** ^***^	0.745^***^	0.500				
s953	440	772	0.833^***^	0.833^***^	0.860^***^	**0.905** ^***^	0.722^***^		0.901			
c5315	2378	4242	0.806^***^	0.836^***^	0.806^***^	**0.836** ^***^	0.644^***^					
imb01	12,732	34.862	**0.868** ^***^	**0.868** ^***^	**0.868** ^***^	**0.868** ^***^	0.619^***^				0.588	0.805

The order and size of each network is also reported. Rent exponents obtained using regression with the best coefficient of determination are in bold. Significance levels of 1% and 5% are marked with (^***^) and (^**^) respectively when using box counting method. The *p*-values are given in Supplementary Information (see Table S1).

To determine the transitions between Region I and Regions II and III, we generated some random networks with the same degree distribution, denoted by (SA). This was done in NetworkX^35^ using the Markov chain scheme proposed by Gkantsidis *et al*.^36^. Applying 20 × *E* double-edge swaps^37^ to each of the networks above, we select a sample of 50 networks with the same degree distribution. The transition between Region I and Region II is determined from some threshold limiting the dispersion in the log-average number of external edges for partitions into boxes of the same size. The transition from Region III to Region I appears when the coefficient of determination of the fitting can no longer be improved above another threshold.

### Experimental results

To compute the Rent exponent, each logic circuit must be partitioned, and the obtained modules must be analyzed. For each partition, the average module size and the average number of external connections per module are calculated representing a data point in a log-log plot. A linear (OLS) regression is then applied to find the slope of the fitted line, which is precisely the Rent exponent of the circuit. For each empirical network, the same method is applied to compute its Rent exponent. Firstly, we implemented the greedy coloring algorithm proposed by Song *et al*.^16^ and described by Concas *et al*.^17^ to obtain a sequence of partitions into boxes of size *b* (i.e. finite sets of nodes of diameter ≤ *b* − 1) varying from 1 to the first integer *b* such that every module of size *b* reduces to the whole vertex set. A *raw* Rent exponent *p* is then estimated using ordinary least squares regression on the data gathered from the above algorithm. The main limitation of this method appears when applied to networks with low diameter, since they are rapidly covered by a single box. Standard errors in the fit of Rent’s rule (1) have been computed and included in Table 2. Naturally, these errors decrease as the diameter of network increase. There is another source of possible error in the estimation of *p* which is related to random choices in the construction of the partitions. However, low values of the standard deviation have been reported by Concas *et al*.^38^ and corroborated by our own numerical experiments.

**Table 2:** Standard error and coefficient of determination in the fit of the power law for different thresholds defining Regions II and III. Best results are in bold.

Network	raw regression		*c* ≥ 0.1		*c* ≥ 0.075			
	std err	R-squared	std err	R-squared	std err	R-squared	std err Δ > 10^−3^	R-squared Δ > 10^−3^
PG	0.058	0.580	0.016	0.993	0.016	0.993	**0.009**	**0.998**
I2	0.060	0.788	**0.027**	**0.990**	**0.027**	**0.990**	**0.027**	**0.990**
CE	0.318	0.131	0.098	0.967	**0.019**	**1.000**	**0.019**	**1.000**
Y2	0.140	0.202	0.030	0.992	0.030	0.992	**0.031**	**0.994**
s838	0.125	0.632	**0.093**	**0.900**	**0.093**	**0.900**	**0.093**	**0.900**
s9234	0.102	0.046	0.026	0.986	0.026	0.986	**0.022**	**0.991**
s953	0.225	0.017	0.026	0.993	0.025	0.995	**0.012**	**0.999**
c5315	0.094	0.544	0.023	0.993	0.023	0.993	**0.023**	**0.995**
ibm01	0.118	0.599	**0.009**	**0.999**	**0.009**	**0.999**	**0.009**	**0.999**

**Figure 1:**
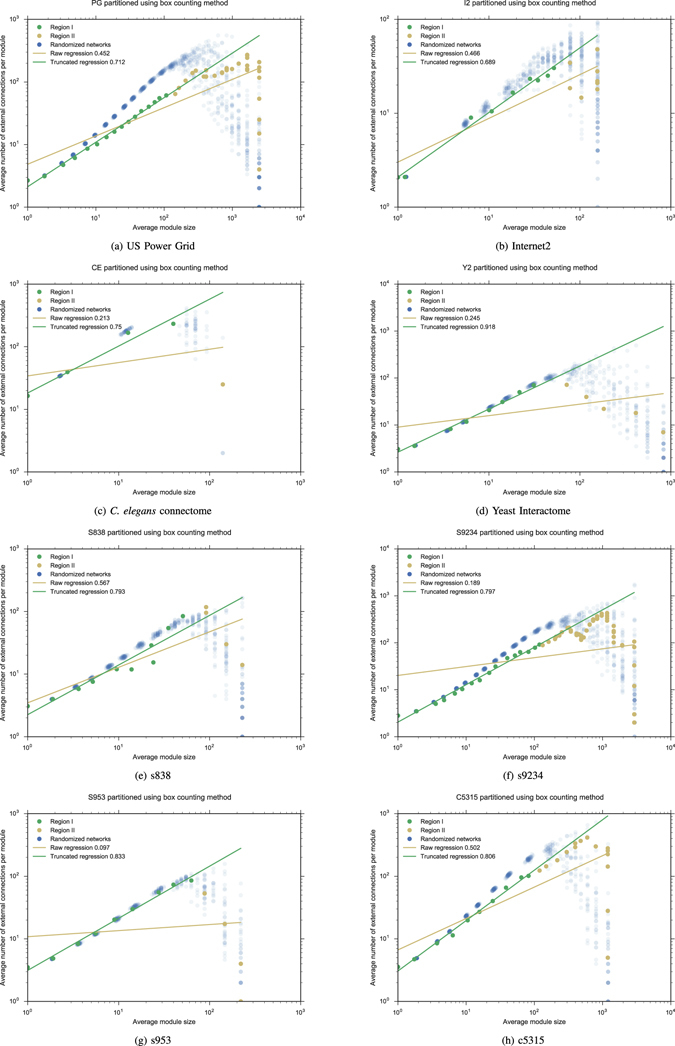
The box counting method is applied to procure the Rent characteristic of each network, as well the whole Rent characteristic of a sample of 50 random networks with the same degree distribution. The Rent exponent obtained by a raw regression is compared with the Rent exponent determined after truncate Region II when the coefficient of variation *c* in the Rentian scaling of the sample is greater than or equal to 0.1. Different results using *c* ≥ 0.075 have been already reported^9^ (see also Table 1).

After generating random graphs with the same degree distribution (using a swap algorithm^37^ according to the scheme proposed by Gkantsidis *et al*.^36^), each of the 50 elements in the sample was partitioned, and the whole data set was represented in a log-log plot. In order to state a criterion for defining Region II, we use the dispersion in the log-average number of external edges for partitions into boxes of the same size. To measure this dispersion, we use the *coefficient of variation c* of the corresponding distribution. Region II is now characterized by the condition *c* ≥ *β* where *β* is a small positive number. Given a fixed value of *β*, the Rent characteristic of the empirical network split into two Regions I and II. Finally, a new regression is applied to find the slope of the fitted line for Region I. Rent characteristics and Rent exponents of the networks PG, I2, CE, Y2, s838, s9234, s953 and c5315 for *β* = 0.1 are exhibited in Fig. 1. See also Fig. 5a for the VLSI circuit ibm01 from the ISPD98 suite. Some values we reported in ref. 9 were a bit higher, obtained with *β* = 0.075. They are also reported in Table 1. The effectiveness of our method for determining Region I and validating Rent’s rule is measured by the standard error and the coefficient of determination *R* ^2^ for the new fits (see Table 2). Significance levels lower than 1% and 5% are also pointed out in Table 1 when using the box counting method, while the exact *p*-values for the different thresholds are reported in Table S1 of Supplementary Information. For fractal networks, like the US Power Grid network^39^, the renormalization procedure described in refs 14, 15 provide a well fitting of the Rent characteristic, even if the boundary effect related to the finiteness of the network is obviously present.

As we can see in Figs 1 and 5a, Region III proposed by Stroobandt^10^ is visible for some VLSI circuits and real networks, but in general it is small (and even absent in some cases) and there is no appreciable variability for the randomized networks associated to the empirical ones. To avoid Rent’s rule underestimates the average number of external connections for low values of *B*, we included in Region III those ranges of values such that the error for the average number of external connections (when we compare empirical data with estimated ones) is positive and the increment Δ in the coefficient of determination *R* ^2^ is always greater than some number *ρ* > 0 when they are successively suppressed. Applying this criterion for *ρ* = 10^−3^, we got new Rent exponents, which are also reported in Table 1. See also Figure S1 in Supplementary Information. By definition, this slightly increases the value of the Rent exponent, as well the coefficient of determination *R* ^2^. However, in the next, we will do not be interested in Region III.

## Rent’s rule from recursive bipartitioning

For the reasons above explained (see ref. 5 for a more detailed discussion), many authors used the partitioning tool hMetis^25^ to study Rent’s rule for both VLSI circuits and biological networks^3, 5, 7, 8, 11, 34, 40^.

### Recursive bipartitioning of randomized networks

**Figure 2:**
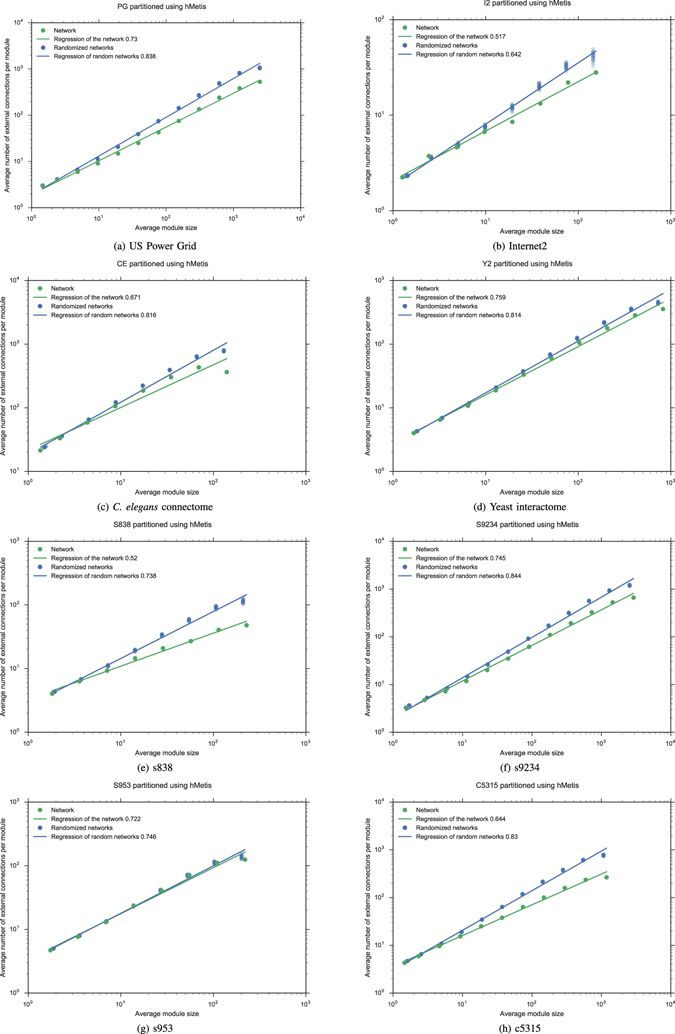
Recursive bipartitioning is applied to procure the Rent characteristic of each network, as well the whole Rent characteristic of a sample of 50 random network with the same degree distribution. Regressions and Rent exponents of both empirical and random networks are compared.

We also used hMetis to describe the Rent characteristic of each network studied here, as well as those of the 50 random graphs in each sample with the same degree distribution. Rent exponents has been estimated, using the same regression method on the data gathered from hMetis, and reported in Table 1. See also Figs 2 and 5b. In this case, the *p*-value is always lower than 4.033 × 10^−5^ (see Supplementary Information for the exact values). As was already observed by Reda^3^, in many of these plots, Region II does not clearly exist. Additionally, it seems that there is no difference between VLSI circuits and other networks. We have not introduced here any criteria to determine Region II, but we analyzed other interesting aspects arising from the comparison of the Rent characteristics provided by both algorithms. In contrast with the box counting method, when recursive bipartitioning is applied to the randomized sample, we observed a reduced variability, and a good fit to a line in every log-log plot. We derived a power law relationship for the average number of external connections between randomized and empirical networks

2 $$P^{\prime} =K{P}^{\alpha }$$

which correspond to a linear relationship *p*′ = *αp* for the Rent exponents. For the empirical networks considered here, this new exponent *α* varies between 1.029 ± 0.002 for s953 and 1.437 ± 0.004 for ibm01. A similar phenomenon occurs when using the box counting method, but only in restriction to Region I where Rent characteristic fits to Rent’s rule. Values for both exponents *α* are reported in Table S2 and log-log plots in Figures S2 and S3, see Supplementary Information. However, there is no significant correlation between the exponents derived from both methods.

Anyway, this experiment raises two different issues: Firstly, how to interpret the peculiar properties of Rent characteristics coming from recursive bipartitioning? Secondly, to what extent does Rent characteristics (independently of the method used to collect data) depend on the degree distribution? Next, we focus on the later.

### Degree distribution

To test the influence of the degree distribution on the Rentian scaling, we added some other random models to the model (SA) based on the swapping algorithm. The *configuration model* (CM) generates (in an equiprobable way) random networks which have a prescribed degree distribution^26, 27^. It can be done in NetworkX^35^ using a random graph generator^41^ based on the approach of Newman^27^. Even starting from the degree sequence of network without multiple or self-connections, it naturally creates networks with this kind of connections. We could reject those samples which have multiple or self-connections until the algorithm gives us a network without multiple or self-connections, but we need to be sure that we are uniformly sampling^41^. However, there is an alternative model (CLM) proposed by Chung and Lu^28^ having the advantage of providing networks without multiple or self-connections. According to this approach, which generalizes Erdös-Rényi model, we can generate random networks with a prescribed sequence of expected degree. To obtain this second model, we used another graph generator^42^ proposed by Miller and Hagberg^43^. Finally, to test the influence of the degree distribution on the Rentian scaling of each network, we considered the largest connected component of the classical Erdös-Rényi model (ER) with the same order *N* and size *E*. We used an implementation^44^ based on the paper by Erdös and Rényi^45^. To a more accurate analysis of the results, all the degree distributions of the empirical graphs, as well those of the different random models (SA), (CM), (CLM) and (ER) are represented in Figure S4, as part of Supplementary Information.

**Figure 3:**
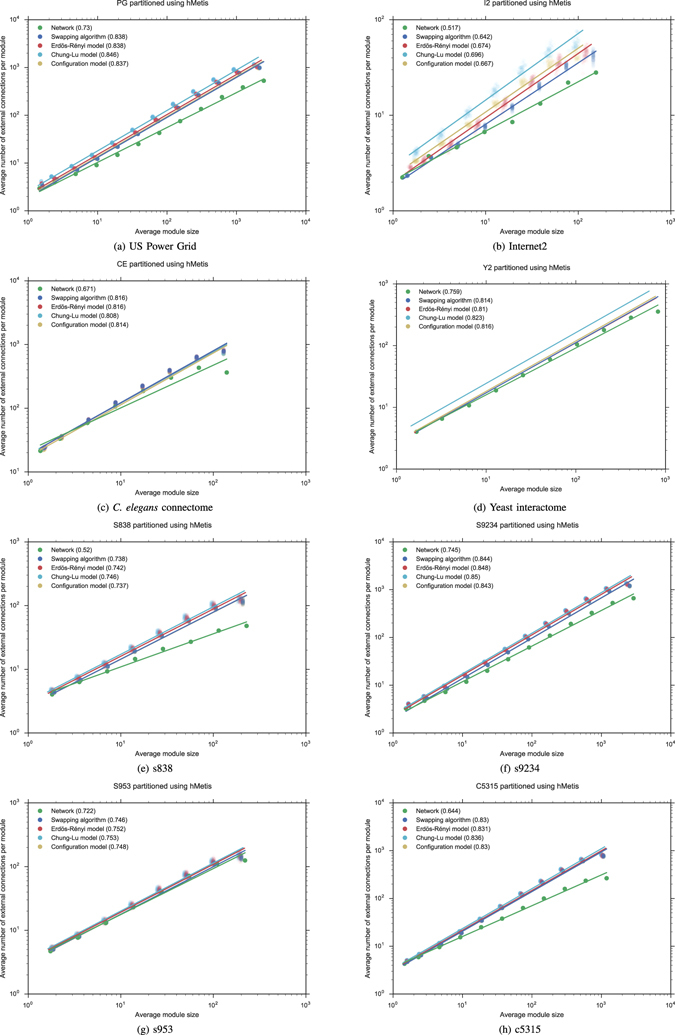
Recursive bipartitioning is applied to obtain the Rent characteristics of each empirical network and each random network (SA), (CM), (CLM) and (ER) with the same order and size.

**Figure 4:**
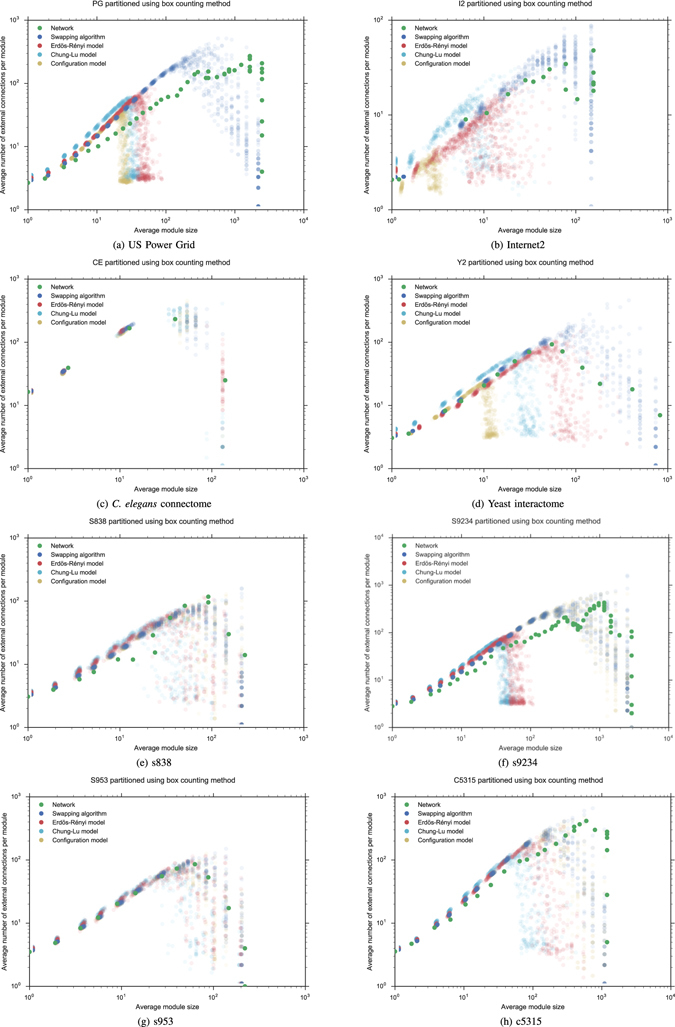
The box counting method is applied to obtain the Rent characteristics of each empirical network and each random network (SA), (CM), (CLM) and (ER) with the same order and size.

**Figure 5:**
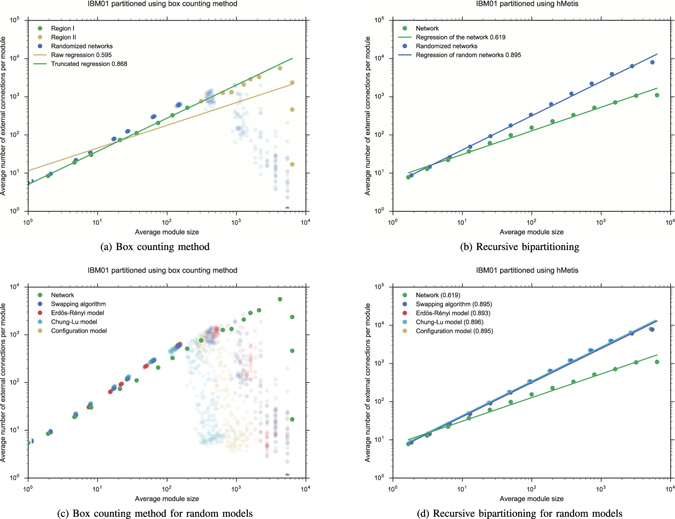
(**a**,**b**) Rent characteristics of the ISPD98 circuit ibm01 with 12,752 nodes and 50,566 edges, which are obtained using the box counting method and recursive bipartitioning. (**c**,**d**) Rent characteristics for randomized networks derived from the ISPD98 circuit ibm01.

Although the Rent exponent of each empirical graph is modified by each randomization procedure, the Rent exponents of the random networks (SA), (CM), (CLM) and (ER) are practically identical in mean (with variations smaller than 0.01 in most cases, except for I2 with a total variation of 0.054 and Y2 with 0.013) independently of whether they have the same distribution or not. The whole Rent characteristics are shown in Figs 3 and 5d. As before, when we replace hMetis with the box counting method, a similar phenomenon can be observed in Figs 4 and 5c, although now the Rent characteristic differs for each random model. In particular, each Rent curve fits to a different Rent’s rule along a different Region I. However, to show the full distribution of the Rentian scaling of the randomized networks, we computed the Rent exponent of each random network in the families (SA), (CM), (CLM) and (ER) and represented the corresponding data in a violin plot (see Figures S5 and S6 in Supplementary Information). It allowed us to observe much more variability in the Rentian scaling computed using the box counting method. Moreover, the *C. elegans* connectome, the yeast interactome and the VLSI circuit s953 also seem show a different behaviour with respect to the randomization procedures.

To avoid the effect of the scales in Figs 3, 4 and 5, we used the exponent of the power law (2) (see Table S2 in Supplementary Information) as a possible measure of the sensitivity with respect to the randomization procedure. Despite the differences related to the partitioning method, the *C. elegans* connectome behaves differently from the US Power Grid network or the academic backbone network Internet2. The last two networks are known to have a high *Q-modularity* ^46, 47^ in consonance with the existence of a central core which is made up of hubs^9^. Thus, using the *Louvain method* ^48^ proposed by Blondel *et al*.^49^, we estimated the modularity *Q* for the whole network set. Table 3 shows the values of *Q* for these networks. It should be noted that most VLSI circuits show a high *Q*-modularity, similar to that of Internet2. The sensitivity with respect to (swapping) randomization is well correlated with the *Q*-modularity when using the box counting method (with Kendall rank correlation coefficient *τ* = 0.778 and *p*-value 0.004 compared with the critical value *τ* = 0.500 for a significance level of 0.05), whereas it is very poorly correlated for recursive bipartioning (with Kendall’s *τ* = 0.167 and *p*-value 0.532). Finally, the Kendall rank correlation between *Q*-modularity and Rent exponent (computed using the box counting method) is also very poor for *c* ≥ 0.1, but it becomes moderately correlated (with Kendall’s *τ* = −0.444 and *p*-value 0.095) for *c* ≥ 0.075. We also notice that for the networks considered here, the *Q*-modularity is well correlated with the average path length *L* (with Kendall’s *τ* = 0.833 and *p*-value < 0.002).

**Table 3:** VLSI circuits and other networks listed by their modularity (in decreasing order) estimated using the Louvain algorithm^48^ for community detection.

Network	Q
PG	0.935
s9234	0.891
imb01	0.865
I2	0.859
c5315	0.835
s838	0.807
Y2	0.739
s953	0.595
CE	0.406

## Discussion

Here we proposed a method to determine Region I where experimental scaling data (comparing the average number of external connections per module with the average number of nodes in each module of a partition) fit well to Rent’s rule. Firstly, we observed that the data sets determined by samples of random networks with the same degree distribution exhibit a certain bifurcation phenomenon. We fixed the transition between Region I and Region II from some threshold in the bifurcation. We tested our method on a set of VLSI circuits and real networks.

However, the scaling curves obtained using the partitioning tool hMetis exhibit a completely different behavior to those given using the box counting method. They show a very small Region II if present at all, a good fit to a line along this large region, and a very reduced variability within the random sample. How to justify these particular properties of the Rentian scaling obtained from the partitioning tool hMetis is a question that would be interesting to answer in the future. Nevertheless, independently of the partitioning method, we also observed that the relationship between the average number of external connections of random models and empirical networks follows a power law with scaling exponent *α* varying between ≈1 and 1.5.

A possible influence of degree distributions on Rent characteristics has been also tested for both box counting and recursive bipartitioning methods. We analyzed Rentian scalings of three different random samples having he same number of nodes and connections than empirical ones. Two variants of a configuration model respecting the degree and the expected degree of each node, together with a classical Erdös-Rényi model, have been added to the initial random model (based on swapping edges) in order to test this influence. We have seen that, in most cases, all these randomization procedures increase the Rent exponent of the randomized network on average. Although there is no substantial difference between the Rent exponents of the different random models when using recursive bipartitioning, the *C. elegans* connectome, the yeast interactome and the VLSI circuit s953 behave differently from the others networks when analyzing the full distribution of the Rentian scalings given by the box counting method. Moreover, there are network architectures more sensitive to the randomization procedures used in the paper, like those of the US Power Grid network or the academic backbone Internet2, both characterized by the existence of a hub core. What architectural properties affect the Rentian scaling of a network and the behavior against random alterations of its structure is another natural question that arises from our work. For the moment, we know that there are differences in the modularity and the sensitiveness of Rentian estimates for the technological networks, including most VLSI circuits, and the biological networks studied here. Both properties are also highly positively correlated (with Kendall’s tau *τ* = 0.778 for *p* = 0.004).

To dispose of an adequate notion of wiring complexity for a biological or technological network, it is essential to be able to correctly interpret the effect of partitioning method on the estimation of the wiring length of the network. Some effort has been done in this direction by several authors in the domain of technological networks^11, 12^, but only recursive bipartitioning^3, 5, 8^ and spectral-based methods^4, 6^ have been used for the biological ones. Analyzing the effect of random alterations on Rent characteristics, we showed some substantial analogies and differences between box counting and partitioning-based methods. We also have achieved some early advances in the direction of detecting some relationship between structural properties and wiring complexity of biological and technological networks, that should be contrasted by analyzing and comparing the Rent characteristics of other biological networks, specifically brain networks.

## Electronic supplementary material

Supplementary Information
