# Supplementary material for: A method for validating Rent’s rule for technological and biological networks
Source: Sci Rep. 2017 Jul 14;7:5378. doi: 10.1038/s41598-017-05670-w (PMC5511203; doi:10.1038/s41598-017-05670-w)
Supplement: Supplementary file 1 — Supplementary Information [file 41598_2017_5670_MOESM1_ESM.pdf]

A method for validating Rent's rule for technological and biological networks – Supplementary information

Fernando Alcalde Cuesta[1,2], **Pablo González Sequeiros**[1,3], **and Álvaro Lozano Rojo**[1,4,5]

[1]GeoDynApp - ECSING Group (Spain)
[2]Departamento de Matemáticas, Universidade de Santiago de Compostela, E-15782 Santiago de Compostela (Spain)
[3]Departamento de Didácticas Aplicadas, Facultade de Formación do Profesorado, Universidade de Santiago de Compostela, Avda. Ramón Ferreiro s/n. E-27002 Lugo (Spain)
[4]Centro Universitario de la Defensa, Academia General Militar, Ctra. Huesca s/n. E-50090 Zaragoza (Spain)
[5]Instituto Universitario de Matemáticas y Aplicaciones, Universidad de Zaragoza, E-50009 Zaragoza (Spain)

First observed by Rent in 1960 and later described by Landman and Russo[1] in the context of logic circuits, *Rent's rule* is a power law relationship between the average number of nodes B for a topological partition of a network and the average number of edges P connecting different modules of the partition, namely

$$P = kB^p \tag{1}$$

where k is the average number of edges per module and p is the *Rent exponent* relating the average number of external connections to the average number of nodes in a log-log scale. Both quantities are directly related by an empirical discrete function

$$P = P_c(B) \tag{2}$$

called *Rent characteristic* by some authors.[2,3] As showed by Landman and Russo,[1] there is an empirical confirmation about the existence of a region, called *Region I*, where Rent characteristic fits well to Rent's rule.

In the paper, we studied Rent's rule for two technological networks (the US Power Grid (PG) network[4] and the academic Internet2 (I2) network[5]), two biological networks (the *C. elegans* neuronal (CE) network[6] and the yeast Protein-Protein Interaction (Y2) network[7]), and five benchmark VLSI circuits (s838, s9234, s953 and c5315 from the ISCAS89 suite[8] and ibm01 from the ISPD98 suite[9]).

S1 Region III

It is known that Rent's rule overestimates the interconnection complexity of the circuit in another region, called *Region II*, where the number of modules is small.[1] But it is was also observed by Stroobandt[10] that Rent's rule underestimates the interconnection complexity of some circuits when the number of modules is large, leading to a new *Region III*. As explained in the paper, we included in Region III those ranges of values such that the error for the average number of external connections (when we compare empirical data with estimated ones) is positive and the increment Δ in the coefficient of determination R^2 is always greater than some number $\rho > 0$ when they are successively suppressed. Applying this criterion for $\rho = 0.001$, we got new transitions between Region I and Regions II and III giving rise to new Rent exponents in some cases (see Table 1 of the paper). For the networks PG, I2, CE, Y2, s838, s9234, s953 and c5315, theses regions and the whole Rent characteristics are shown in Figure S1 below.

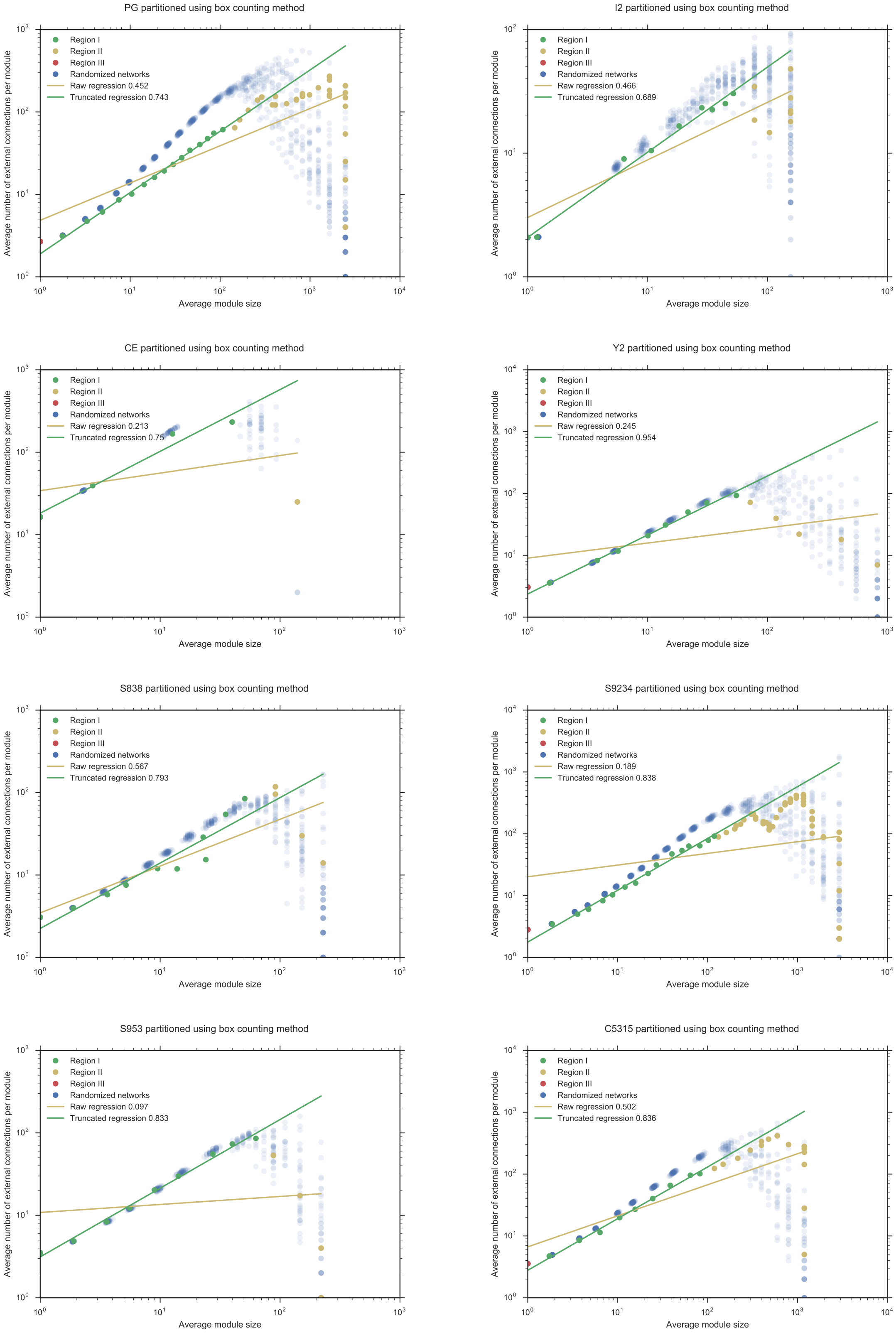

Figure S1. Region III for $\Delta > 0.001$.

S2 Fitting Rent's rule to the data set

Standard errors and coefficients of determination for fitting Rent's rule to data are detailed in the paper (see Table 2), but significance levels lower than 1% and 5% have been also pointed out in Table 1. Here we detail the exact p-values when using the box counting method:

Table S1. The p-values for fitting Rent's rule using the box counting method and different thresholds.

Network	Raw	$c \geq 0.1$		$c \geq 0.075$		hMetis
		No Reg. III	$\Delta > 10^{-3}$	No Reg. III	$\Delta > 10^{-3}$	
PG	7.995×10^{-10}	1.313×10^{-16}	8.023×10^{-19}	1.313×10^{-16}	8.023×10^{-19}	8.027×10^{-14}
I2	9.063×10^{-7}	3.228×10^{-8}	4.569×10^{-5}	3.228×10^{-8}	4.569×10^{-5}	2.201×10^{-7}
CE	0.551	0.017	0.168	0.013		4.033×10^{-5}
Y2	0.107	1.116×10^{-8}	8.203×10^{-8}	1.116×10^{-8}	8.203×10^{-8}	1.096×10^{-10}
s838	6.732×10^{-4}	2.829×10^{-5}	1.245×10^{-4}	2.829×10^{-5}	1.245×10^{-4}	3.267×10^{-7}
s9234	0.0680	2.731×10^{-14}	1.053×10^{-14}	2.731×10^{-14}	1.053×10^{-14}	6.403×10^{-14}
s953	0.675	7.643×10^{-9}	1.033×10^{-7}	4.464×10^{-8}	6.417×10^{-9}	6.836×10^{-7}
c5315	1.719×10^{-5}	5.114×10^{-10}	2.752×10^{-9}	5.114×10^{-10}	2.752×10^{-9}	1.583×10^{-11}
imb01	1.003×10^{-4}	3.468×10^{-12}	5.403×10^{-12}	3.468×10^{-12}	3.167×10^{-9}	5.746×10^{-12}

S3 Comparing external connections for randomized and empirical networks

As observed in the paper, when recursive bipartitioning is applied to the randomized sample, we have a reduced variability, and a good fit to a line in every log-log plot (see Figure 2). We derived a power law relationship for the average number of external connections between randomized and empirical networks

$$P' = KP^{\alpha} \tag{3}$$

which correspond to a linear relationship $p' = \alpha p$ for the Rent exponents. For the empirical networks considered in the paper, this new exponent α varies between 1.029 ± 0.002 for s953 and 1.437 ± 0.004 for ibm01. A similar phenomenon is visible using the box counting method, but only in restriction to Region I where Rent characteristic fits to Rent's rule (see Figure 1). Values for both exponents α are reported in Table S1 and log-log plots in Figures S2 and S3. All the p-values are essentially 0.

Table S2. Exponent in the power law (3) comparing Rentian scaling of empirical graphs and swapping randomized models.

Network	Box counting	hMetis
PG	1.491 ± 0.006	1.147 ± 0.002
I2	1.125 ± 0.011	1.229 ± 0.006
CE	0.978 ± 0.016	1.178 ± 0.007
Y2	1.127 ± 0.005	1.071 ± 0.001
s838	1.008 ± 0.011	1.413 ± 0.006
s9234	1.354 ± 0.005	1.132 ± 0.000
s953	0.991 ± 0.007	1.029 ± 0.002
c5315	1.233 ± 0.005	1.288 ± 0.002
imb01	1.141 ± 0.005	1.437 ± 0.004

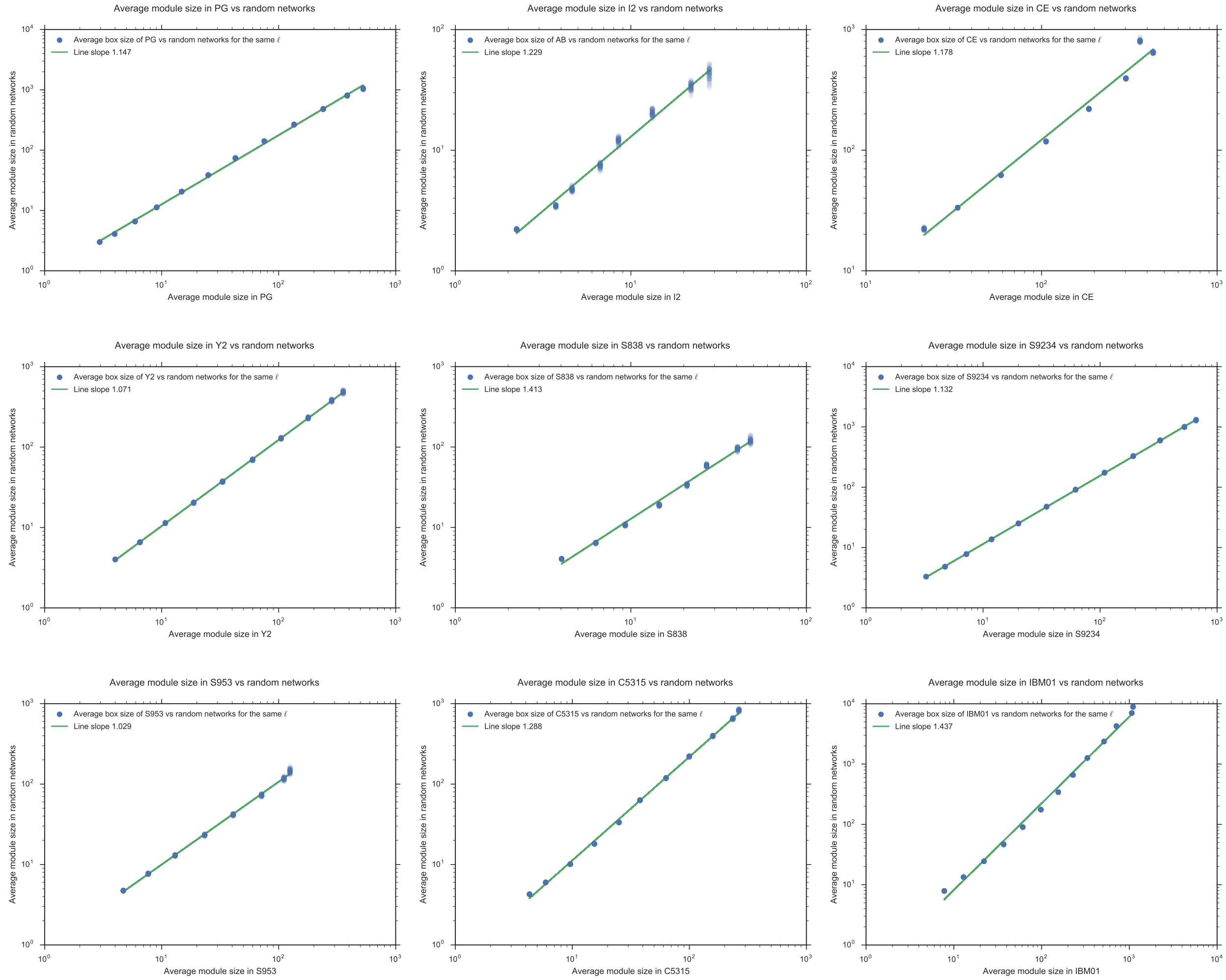

Figure S2. Power law relationship for the average number of external connections between randomized and empirical networks using the partitioning tool hMetis.

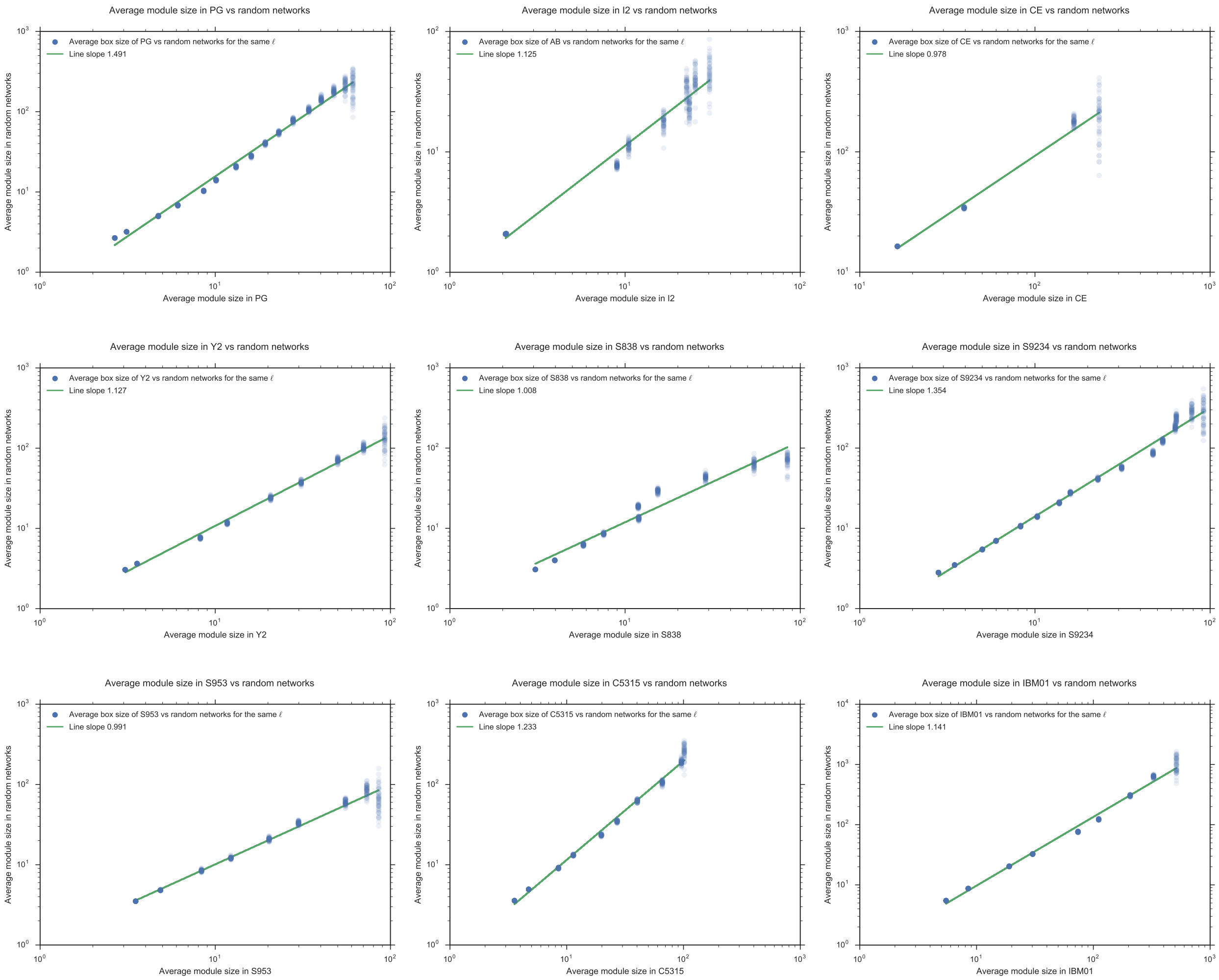

Figure S3. Power law relationship for the average number of external connections between randomized and empirical networks using the box counting method.

S4 Degree distributions

In the paper, to test the influence of the degree distribution on the Rentian scaling, we consider some random models other than the model (SA) based on the swapping algorithm and used to validating Rent's rule. The *configuration model* (CM) generates random networks which have a prescribed degree distribution,[11,12] but multiple or self-connections may appear. For the empirical networks above listed, it was be done in NetworkX[13] using a random graph generator[14] based on the approach of Newman.[12] There is another model (CLM), proposed by Chung and Lu,[15] which generates random networks (having no multiple or self-connections) with a prescribed sequence of expected degree. In this case, we used a graph generator[16] proposed by Miller and Hagberg.[17] Finally, we considered the largest connected component of the classical Erdös-Rényi model (ER) with the same order N and size E according to an implementation[18] based on the original paper by Erdös and Rényi.[19] Their degree distributions are compared in Figure S4 below.

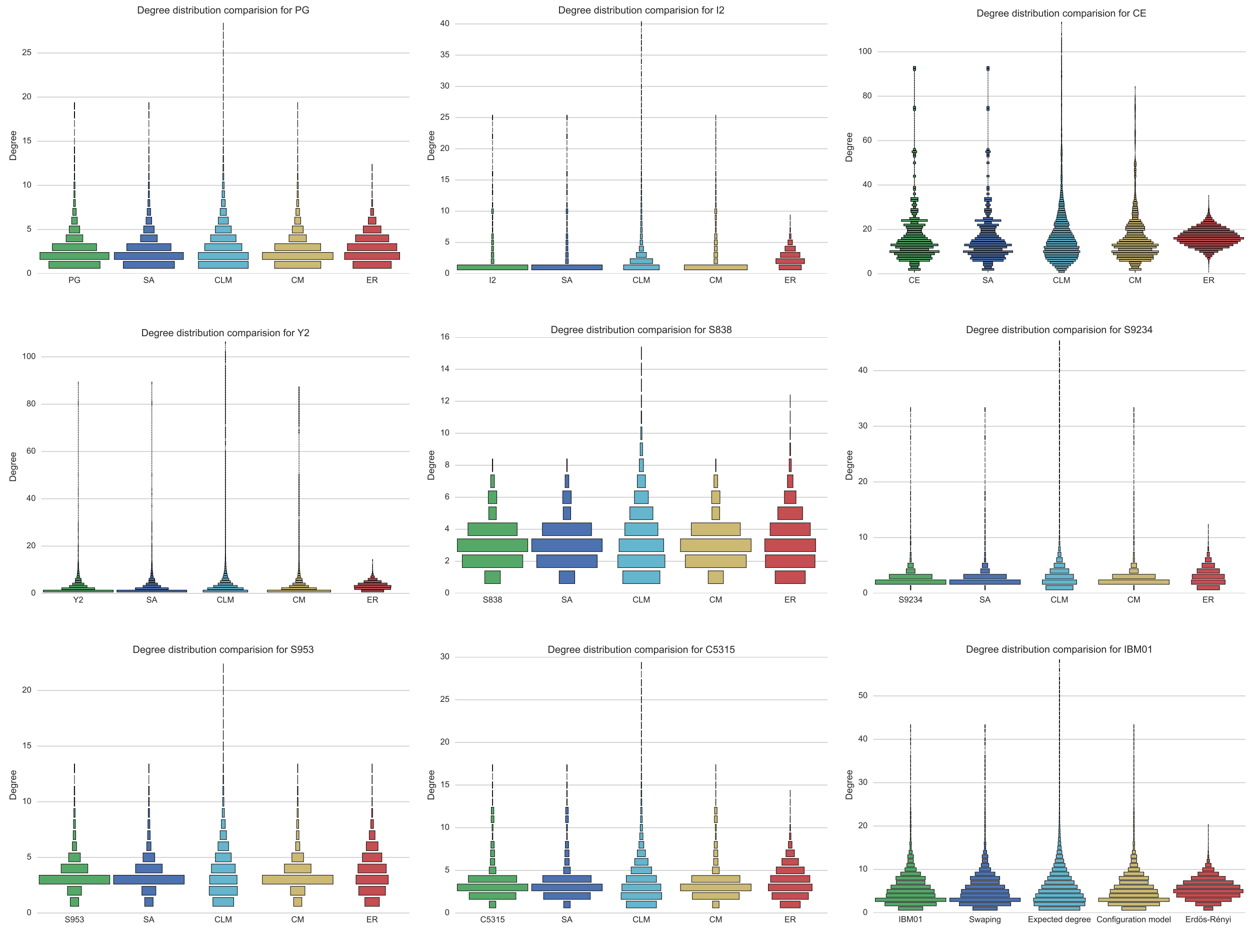

Figure S4. Degree distribution of each empirical network and each random model (SA), (CM), (CLM) and (ER) with the same order and size.

S5 Rentian scalings for randomized networks

The distribution of the Rentian scaling for the random networks in the families (SA), (CM), (CLM) and (ER) has been described by computing the Rent exponent of all these networks. The well-fitted regions were fixed from the bifurcation phenomena observed in each family (see Figure 3). Note that there is no good fitting for the random networks obtained from the academic backbone network Internet2[20] when using the configuration model (CM) and the Erdös-Rényi model (ER). Violin plots have been used in order to visualize clearly the distribution of Rent exponents in Figures S5 and S6.

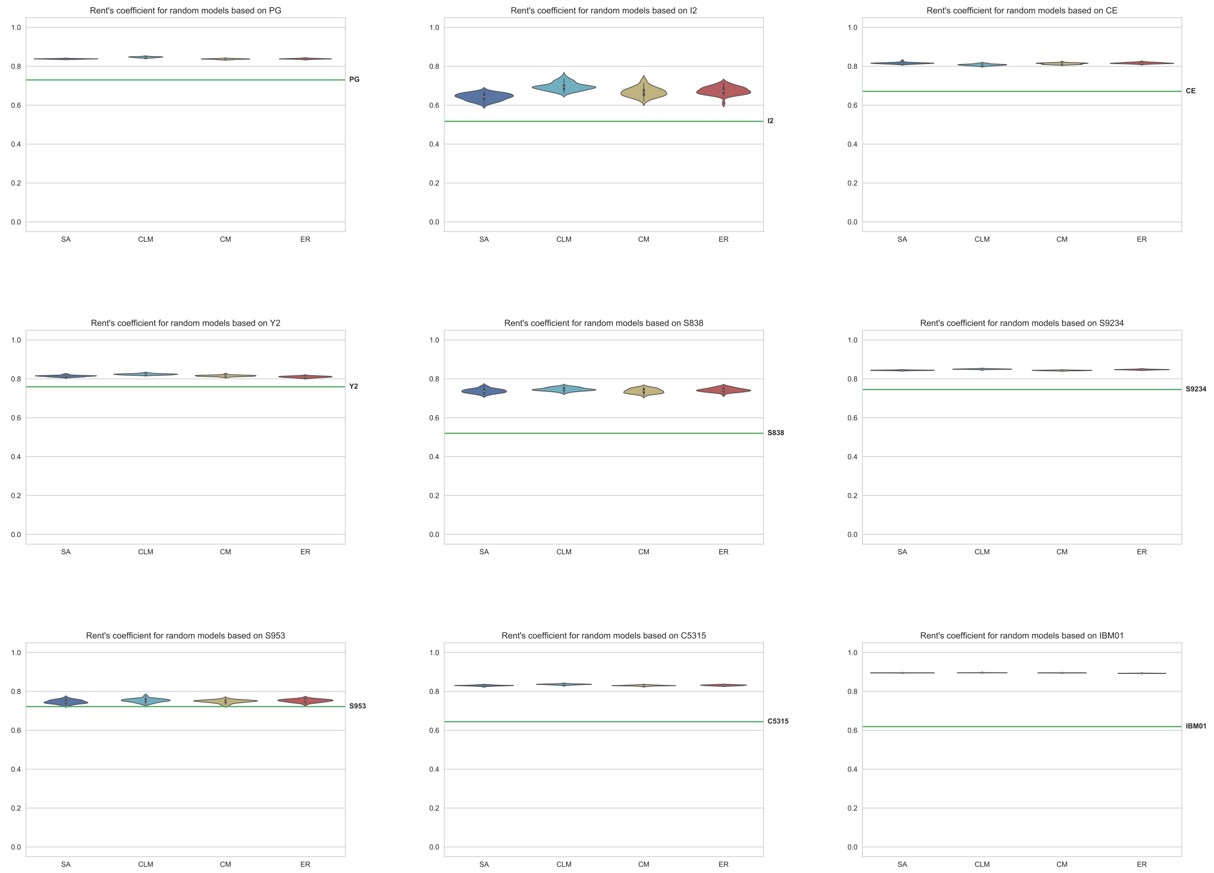

Figure S5. Distribution of Rent exponents of random networks associated to each empirical network according to the models (SA), (CM), (CLM) and (ER) by using the recursive bipartioting tool hMetis.

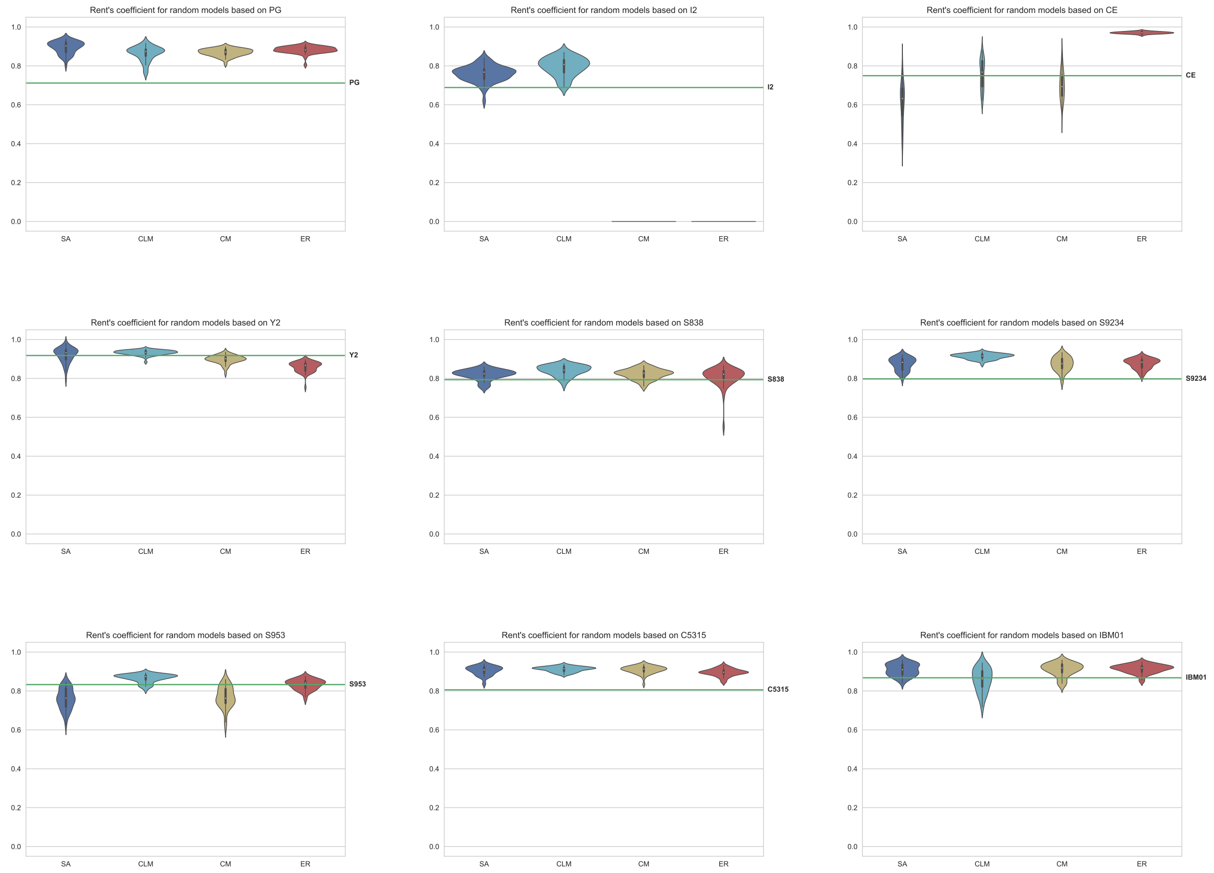

Figure S6. Distribution of Rent exponents of random networks associated to each empirical network according to the models (SA), (CM), (CLM) and (ER) by using the box counting method.

S6 Modularity

The notion of Q-modularity was introduced by Newman and Girvan[21] to measure the decomposability of a network into modules. Given a partition $\mathscr{P}$ of the vertex set $V = \{1,\dots,N\}$ of an undirected connected network $G = (V,E)$, the modularity of $\mathscr{P}$ is given by

$$Q = \frac{1}{2|E|}\sum_{M\in\mathscr{P}}\sum_{i,j\in M}\left(a_{ij} - \frac{d_i d_j}{2|E|}\right),$$

where a_{ij} is the entry of the adjacency matrix corresponding to two nodes i and j in some module $M \in \mathscr{P}$ having degrees d_i and d_j. Several algorithms were proposed to detected the modular structure of the network by finding partitions with the largest value of Q. See a comparative analysis in Ref. 22. Other methods to study hierarchical modularity have been introduced from the idea of decomposing modules into submodules, which in turn are decomposed into sub-submodules, and so on. Proposed by Blondel et al.,[23] the Louvain method[24] takes advantage of the hierarchical structure of the network to accelerate the optimization of Q. In Figure S7 below, we can see how the different randomization procedures affect to the modularity of the empirical networks considered in the paper.

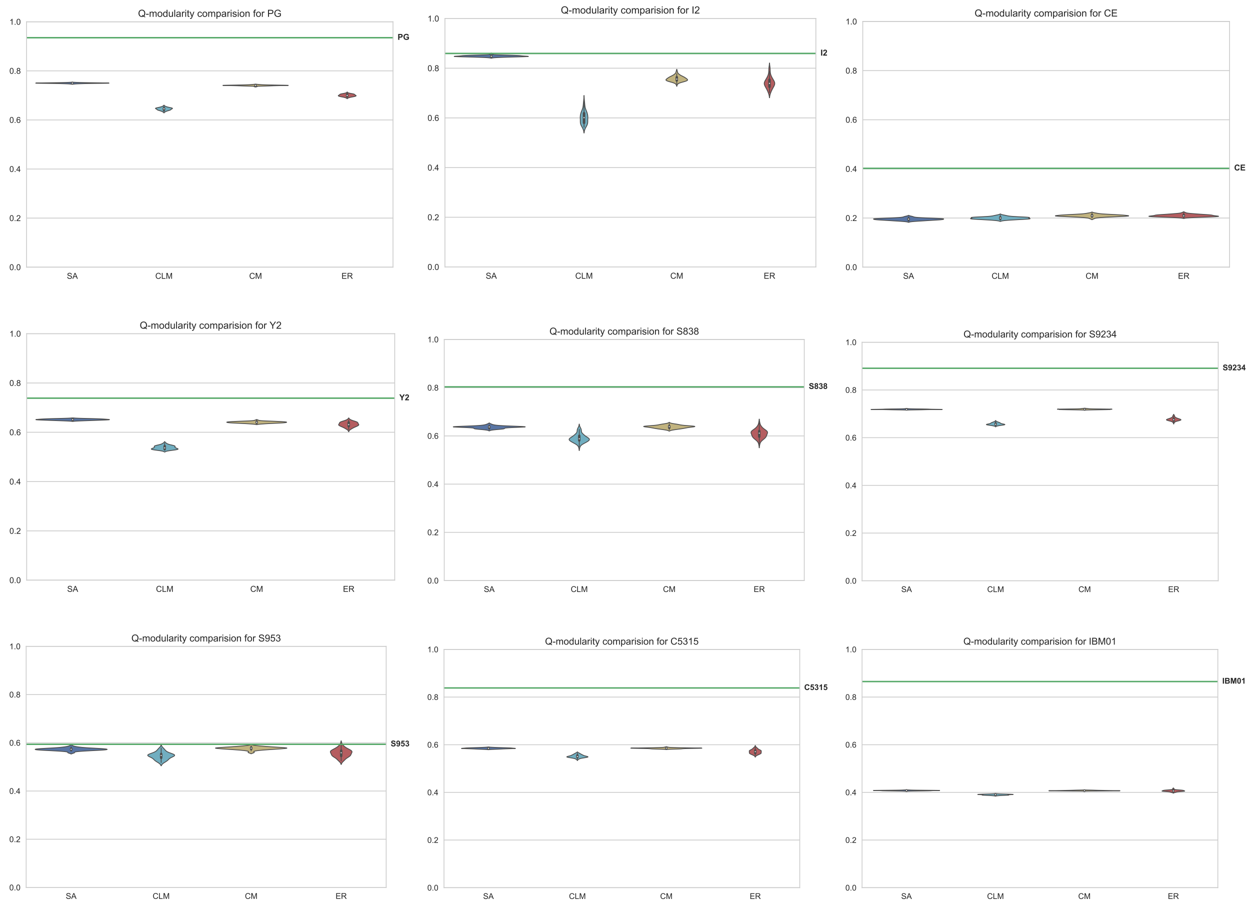

Figure S7. Modularity of the random models (SA), (CM), (CLM) and (ER) compared with modularity of each empirical network.

References

1. Landman, B. & Russo, R. L. On a pin versus block relationship for partitions of logic graphs. *IEEE Transactions on Computers* **C-20**, 1469–1479 (1971).

2. Stroobandt, D., Verplaetse, P. & van Campenhout, J. Generating synthetic benchmark circuits for evaluating CAD tools. *Trans. Comp.-Aided Des. Integ. Cir. Sys.* **19**, 1011–1022 (2006).

3. Partzsch, J. & Schüffny, R. Developing structural constraints on connectivity for biologically embedded neural networks. *Biological Cybernetics* **106**, 191–200 (2012).

4. Koblenz Network Collection. U.S. Power Grid Network Dataset. Available at: http://konect.uni-koblenz.de. Accessed: April 3th, 2015.

5. GlobalNOC. *Internet2 Maps & Documentation*. Available at: http://noc.net.internet2.edu/i2network/maps-documentation.html. Accessed: April 3th, 2015.

6. Varshney, L. R., Chen, B. L., Paniagua, E., Hall, D. H. & Chklovskii, D. B. *Neuronal connectivity II*. Available at: http://www.wormatlas.org/neuronalwiring.html. Accessed: April 15th, 2015.

7. Interactome Projects at CCSB. *Yeast Interactome Project*. Available at: http://interactome.dfci.harvard.edu/S_cerevisiae/index.php. Accessed: September 3th, 2015.

8. Brglez, F., Bryan, D. & Kozminski, K. Combinational profiles of sequential benchmark circuits. In *IEEE International Symposium on Circuits and Systems, 1989*, 1929–1934 vol.3 (1989).

9. Alpert, C. J. The ISPD98 circuit benchmark suite. In *Proceedings of the 1998 International Symposium on Physical Design*, ISPD '98, 80–85 (ACM,New York, NY, USA, 1998).

10. Stroobandt, D. On an efficient method for estimating the interconnection complexity of designs and on the existence of region III in Rent's rule. In *Proceedings of the Ninth Great Lakes Symposium on VLSI*, 330–331 (IEEE, 1999).

11. Molloy, M. & Reed, B. A critical point for random graphs with a given degree sequence. *Random Struct. Algorithms* **6**, 161–179 (1995).

12. Newman, M. E. J. The structure and function of complex networks. *SIAM Review* **45**, 167–256 (2003).

13. Hagberg, A. A., Schult, D. A. & Swart, P. J. Exploring network structure, dynamics, and function using NetworkX. In *Proceedings of the 7th Python in Science Conference (SciPy2008)*, 11–15 (Pasadena, CA USA, 2008).

14. NetworkX. *Configuration model generator*. Available at: http://networkx.readthedocs.io/en/stable/reference/generated/networkx.generators.degree_seq.configuration_model.html. Accessed: November 10th, 2016.

15. Chung, F. & Lu, L. Connected components in random graphs with given expected degree sequences. *Annals of Combinatorics* **6**, 125–145 (2002).

16. NetworkX. *Expected degree graph generator*. Available at: http://networkx.readthedocs.io/en/stable/reference/generated/networkx.generators.degree_seq.expected_degree_graph.html. Accessed: November 10th, 2016.

17. Miller, J. C. & Hagberg, A. *Efficient Generation of Networks with Given Expected Degrees*, 115–126(Springer Berlin Heidelberg, Berlin, Heidelberg, 2011).

18. NetworkX. *Erdös-Rényi graph generator*. Available at: http://networkx.readthedocs.io/en/stable/reference/generated/networkx.generators.random_graphs.erdos_renyi_graph.html. Accessed: November 10th, 2016.

19. Erdös, P. & Rényi, A. On random graphs I. *Publ. Math. Debrecen* **6**, 290–297 (1959).

20. Alcalde Cuesta, F., González Sequeiros, P. & Lozano Rojo, Á. Exploring the topological sources of robustness against invasion in biological and technological networks. *Scientific Reports* **6**, 20666 EP – (2016).

21. Newman, M. E. J. & Girvan, M. Finding and evaluating community structure in networks. *Phys. Rev. E* **69**, 026113 (2004).

22. Newman, M. E. J. Modularity and community structure in networks. *Proceedings of the National Academy of Sciences* **103**, 8577–8582 (2006).

23. Blondel, V. D., Guillaume, J.-L., Lambiotte, R. & Lefebvre, E. Fast unfolding of communities in large networks. *Journal of Statistical Mechanics: Theory and Experiment* **2008**, P10008 (2008).

24. Blondel, V. D. *The Louvain method for community detection in large networks*. Available at: https://perso.uclouvain.be/vincent.blondel/research/louvain.html. Accessed: November 28th, 2016.
